# Occurrence of Highly Conjugative IncX3 Epidemic Plasmid Carrying *bla*_NDM_ in *Enterobacteriaceae* Isolates in Geographically Widespread Areas

**DOI:** 10.3389/fmicb.2018.02272

**Published:** 2018-09-20

**Authors:** Ya Wang, Man-Ki Tong, Kin-Hung Chow, Vincent Chi-Chung Cheng, Cindy Wing-Sze Tse, Alan Ka-Lun Wu, Raymond Wai-Man Lai, Wei-Kwang Luk, Dominic Ngai-Chong Tsang, Pak-Leung Ho

**Affiliations:** ^1^Department of Microbiology, Queen Mary Hospital, Carol Yu Centre for Infection, The University of Hong Kong, Hong Kong, China; ^2^Department of Clinical Pathology, Kwong Wah Hospital, Hospital Authority, Hong Kong, China; ^3^Department of Clinical Pathology, Pamela Youde Nethersole Eastern Hospital, Hospital Authority, Hong Kong, China; ^4^Department of Microbiology, Prince of Wales Hospital, Hospital Authority, Hong Kong, China; ^5^Department of Pathology, Tseung Kwan O Hospital, Hospital Authority, Hong Kong, China; ^6^Department of Clinical Pathology, Queen Elizabeth Hospital, Hospital Authority, Hong Kong, China

**Keywords:** carbapenems, antimicrobial resistance epidemiology, molecular epidemiology, *Enterobacteriaceae*, resistance plasmid

## Abstract

The emergence of New Delhi metallo-β-lactamase (NDM) in common enterobacterial species is a major concern for healthcare. Early reports have revealed that the spread of NDM involved diverse and heterogeneous plasmids. Recently, the involvement of a rare, IncX3 subtype plasmid has been increasingly recognized. Here, we studied the prevalence of IncX plasmid subtypes in 198 carbapenem-resistant *Enterobacteriaceae*, originating from a territory-wide active surveillance in Hong Kong in 2016. The complete sequences and biological features of the *bla*_NDM_-carrying plasmids were investigated. A total of 62 NDM-type, 21 OXA-48 type, 14 IMP-type, 8 KPC-type, 4 IMI-type producers, and 89 non-carbapenemase-producers were tested for presence of IncX subtypes. IncX3 (*n* = 60) was the most common subtype, followed by IncX4 (*n* = 6) and IncX1 (*n* = 2). The prevalence of IncX3 subtype in isolates producing NDM, other carbapenemase types and non-carbapenemase producers were 75.8, 21.3, and 3.4%, respectively (*P* < 0.001). An IncX3 plasmid (size ∼50 kb) was confirmed to carry *bla*_NDM_ in 47 isolates of different enterobacterial species. Thirteen IncX3 plasmids originating from six healthcare regions in Hong Kong were completely sequenced. The results showed that the IncX3 plasmids carrying *bla*_NDM_ share a high degree of sequence identity with a previously reported plasmid, pNDM-HN380 (GenBank accession JX104760), over the backbone and genetic load regions. A blast search further revealed the occurrence of identical or nearly identical IncX3 plasmids carrying *bla*_NDM_ in other part of China, Korea, Myanmar, India, Oman, Kuwait, Italy, and Canada. Two IncX3 carrying *bla*_NDM_ were investigated further. Conjugation experiments demonstrated that the IncX3 plasmids could be efficiently transferred to multiple enterobacterial species at frequencies that are comparable or higher than the epidemic IncFII plasmid carrying *bla*_CTX-M_ (pHK01). In addition, efficient transfer of the NDM plasmids occurred over a range of temperatures. In conclusion, this study demonstrated the important role played by IncX3 in the dissemination of NDM and the occurrence of pNDM-HN380-like plasmids in geographically widespread areas. The high mobility of IncX3 plasmid across different enterobacterial species highlights the ability of this plasmid replicon to be an important vehicle in worldwide dissemination of NDM.

## Introduction

The worldwide dissemination of bacteria producing New Delhi metallo-β-lactamase (NDM) is a major public health concern. NDM was first described for *Klebsiella pneumoniae* from a patient who was previously admitted to a hospital in India in 2009 (Yong et al., 2009). Since then, NDM producing bacteria have been identified in >40 countries (Khan et al., 2017). Many reports have described patients who have visited NDM endemic areas, such as the Indian subcontinent and Balkan states, and then returning home with NDM-producing bacteria causing colonization or infection, and subsequent local spread (Nordmann et al., 2011). Molecular investigations have revealed that the spread of NDM involved complex pathways and a high level of inter-genus, -species, and -lineages gene transfer (Johnson and Woodford, 2013; Khan et al., 2017). Early reports further highlighted the involvement of diverse and heterogeneous NDM-carrying plasmids (Nordmann et al., 2011). However, it is possible that the epidemiology of NDM may change, as the gene may be carried by high risk international bacterial clones and epidemic resistance plasmids (Johnson and Woodford, 2013).

In China, reports of NDM-producing bacteria are on the rise, especially among *Enterobacteriaceae* from hospitalized patients (Yang et al., 2014; Zhang et al., 2016; Dong et al., 2017). In 2012, our group reported the involvement of IncX3 plasmids (represented by pNDM-HN380) in the dissemination of *bla*_NDM-1_ in multiple geographic areas in China (Ho et al., 2012b). Subsequently, pNDM-HN380-like plasmids carrying *bla*_NDM-1_ or variants have also been described in India, the Arabian Peninsula, Europe, and Australia (Wailan et al., 2015; Petrosillo et al., 2016; Riazzo et al., 2017; Choudhury et al., 2018; Ho et al., 2018a). In a recent survey of more than a thousand carbapenem-resistant *Enterobacteriaceae* originating from hospitals located in 25 provinces and municipalities in China, the prevalence of *bla*_NDM_ was found to be 31% (Zhang et al., 2017). Of note, *bla*_NDM_ was found to be harbored on IncX3 plasmids in a large majority (92%) of the *Escherichia coli* and *K. pneumoniae* isolates (Zhang et al., 2017). In South Korea, among 146 NDM-producing *Enterobacteriaceae* recovered from 33 general hospitals in 2010–2015, IncX3 was the predominant plasmid type (77%) harboring *bla*_NDM_ (Yoon et al., 2018).

The IncX plasmids are narrow host range plasmids of *Enterobacteriaceae* (Johnson et al., 2012). These plasmids have been identified mainly from *Salmonella* and *E. coli* from diverse sources at low frequencies of 8.7 to 10.6% (Lo et al., 2014; Dobiasova and Dolejska, 2016). In general, subtypes IncX1, IncX2, and IncX4 have been detected more commonly than IncX3 subtype (Dobiasova and Dolejska, 2016). In this investigation, we studied the prevalence of IncX3 and other subtypes in a collection of carbapenem-resistant *Enterobacteriaceae* (CRE) originating from an active surveillance in Hong Kong. The biological features of the *bla*_NDM_-carrying IncX3 plasmids were investigated.

## Materials and Methods

### Bacterial Isolates and Identification

A total of 198 CRE isolates, collected from 14 hospitals in Hong Kong in 2016 were investigated. The isolates were collected from a CRE surveillance program involving all the healthcare regions (Ho et al., 2011, 2016). This comprised 109 carbapenemase-producing *Enterobacteriaceae* (CPE) and 89 carbapenemase-negative *Enterobacteriaceae* (CNE). The isolates (∼25%) were randomly selected from a total collection of 497 CPE and 331 CNE isolates in that year. Only one isolate from each patient was included. The species distribution for the final collection of CPE and CNE strains were, respectively, as follows: 49 and 30 *E. coli*, 32 and 38 *K. pneumoniae*, 11 and 8 *Enterobacter cloacae*, 6 and 3 *Citrobacter freundii*, 4 and 6 *Enterobacter aerogenes*, 4 and 1 *Klebsiella oxytoca*, and 3 and 3 other *Enterobacteriaceae*. The carbapenemase types in the isolates included NDM (*n* = 62), OXA-48 group (*n* = 21), IMP (*n* = 14), KPC (*n* = 8), and IMI (*n* = 4). The CPE isolates included 19 (17.4%) isolates from clinical specimens (including 3 blood cultures, 9 urines, 5 wounds, 1 bile fluid and 1 tracheal aspirate) and 90 (82.6%) isolates from rectal swabs. All except one CNE were recovered from rectal swabs. The isolates were identified to species level by MALDI-TOF MS (Chen et al., 2013) and were stored in MicroBank (Pro-Lab Diagnostics) at -80°C. All the isolates were retrospectively retrieved for this work.

### Susceptibility Testing

Disk diffusion method was used to determine the susceptibility of the isolates to ertapenem, imipenem, and meropenem (5). All the isolates were resistant to at least one of the carbapenem. Phenotypic expression of carbapenemase in the isolates was confirmed with a strip Carba NP test (Ho et al., 2018b). For the subset of NDM-producing isolates carrying IncX3 plasmids, MICs of the carbapenems and a panel of drugs (amikacin, colistin, gentamicin, levofloxacin, minocycline, and tigecycline) with potential activities were determined by a broth microdilution procedure using Sensititre plates (Thermo Fisher Scientific, West Sussex, United Kingdom). Cation-adjusted Mueller-Hinton broth was used according to the manufacturer’s recommendation. The plates were inoculated and the endpoints read after incubation in ambient air at 35°C for 18 h using a Thermo Fisher Scientific^TM^ Sensitre^TM^ ARIS^TM^ 2X instrument. Quality control was performed with *Pseudomonas aeruginosa* ATCC 27853 and *E. coli* ATCC 25922. EUCAST breakpoints were used for interpretation of colistin and tigecycline MIC (EUCAST, 2018). MICs for the other drugs were interpreted according to the CLSI (CLSI, 2018).

### Molecular Studies

PCR assays were used to detect carbapenemase genes (*bla*_KPC_, *bla*_IMI_, *bla*_IMP_, *bla*_NDM_, *bla*_V IM_, and *bla*_OXA-48_). The allele of the carbapenemase genes were determined by Sanger sequencing (Ho et al., 2016; Kong et al., 2018). The isolates were surveyed for presence of the IncX1 to IncX5 subtypes plasmid replicons using previously described PCR procedures (Lo et al., 2014). The co-transfer of *bla*_NDM_ and the IncX3 plasmid was investigated by filter mating using J53 as the recipient. Transconjugants were selected on Luria-Bertani (LB) agar plates supplemented with sodium azide (100 μg ml^-1^) and meropenem (0.25 μg ml^-1^). Plasmid location of the *bla*_NDM_ and IncX3 replicon were confirmed by hybridization using specific PCR products as probes (Wang et al., 2017). PCR and Sanger sequencing was used to determine the multilocus sequence types (MLST) of *K. pneumoniae* and *E. coli* using the Pasteur Institute and the Warwick scheme, respectively (Diancourt et al., 2005; Wirth et al., 2006).

### Plasmid Sequencing

Thirteen isolates with the *bla*_NDM_ genes carried on IncX3 plasmids were sequenced. The isolates (one from blood culture, one from bile fluid and 11 from rectal swabs) were chosen to provide representation from different hospitals in the collection. The IncX3 plasmids carrying *bla*_NDM_ in the original isolates (*n* = 13) were sequenced by an Illumina HiSeq platform at ∼100-fold coverage. The plasmids were assembled *de novo* using a CLC Genomics Workbench (Qiagen, Redwood City, United States) and gaps were closed by additional PCRs and Sanger sequencing (Wang et al., 2017; Ho et al., 2018a). ISfinder^1^ was used to identify and annotate insertion sequences.

### Conjugation Studies

The biological properties of two *bla*_NDM_-carrying IncX3 plasmids (both approximately 50 kb in size and carrying *bla*_CTX-M_ originating from two human *E. coli* strains CRE 396 and CRE 1493) and the IncFII plasmid pHK01 (70 kb, carrying *bla*_CTX-M-14_ from human *E. coli* isolate combat2D2, GenBank accession HM355591) were compared. pHK01 is an epidemic plasmid which plays an important role in the dissemination of *bla*_CTX-M-14_ among *E. coli* isolates in certain Asian countries (Ho et al., 2012c; Jiang et al., 2017). The frequencies of conjugational transfer of the plasmids to a panel of 11 isolates comprising *P. aeruginosa* (*n* = 3), *Acinetobacter baumannii* (*n* = 2), *E. coli* (*n* = 3, including J53 and two clinical isolates of ST131 and ST405), *Shigella flexneri* (*n* = 1), *Salmonella enteritidis* (*n* = 1), and *K. pneumoniae* (*n* = 1) were investigated. The two clinical *E. coli* isolates were chosen to represent two widespread clones of multilocus sequence types (ST) 131 and 405. In addition, the frequencies of plasmid transfer to J53 recipient was assessed at three different temperatures (30, 37, and 42°C). All conjugation, unless otherwise specified, were performed at 37°C at donor to recipient ratio of 1 to 2. Transconjugants were selected on either MacConkey, XLD, or UriSelect^TM^4 (Bio-Rad, CA, United States) agar plates containing supplemented sodium azide (100 μg ml^-1^), meropenem (0.25 μg ml^-1^) and/or cefotaxime (1 μg ml^-1^) as appropriate (**Supplementary Table S1**). The frequency of transfer was expressed as transconjugants per donor cell (T/D) as previously described (Zhong et al., 2012). Donor and recipient cultures placed separately on filters were included as controls in each run and no growth was observed on the transconjugants selection media. All experiments were carried out four times (biological duplicates and technical duplicates) and mean values presented with standard deviations.

### Statistical Analysis

Proportions were compared using Chi-Square or Fisher exact tests. Conjugation frequencies were logarithmically transformed and compared using one way ANOVA. All analyses were performed using IBM SPSS Statistics, version 24 (Hong Kong). A two-tailed *P*-value of <0.05 was considered as significant.

## Results

### Prevalence of IncX Replicons

A total of 68 IncX replicons were detected in 64 isolates including 2 IncX1, 60 IncX3, and 6 IncX4 subtypes (**Table 1**). There were differences in the relative occurrence of the IncX subtypes among the isolates. The IncX3 subtypes was more commonly found in NDM producers (75.8%, 47/62) than in isolates producing other types of carbapenemase (21.3%, 10/47, *P* < 0.001) and carbapenemase non-producers (3.4%, 3/89, *P* < 0.001).

**Table 1:** IncX replicons among 198 carbapenem-resistant *Enterobacteriaceae* isolates.

IncX subtype^a^	Number of isolates (% by column), according to carbapenemase type			
	NDM (*n* = 62)	Other carbapenemases (*n* = 47)	None (*n* = 89)	Total (*n* = 198)
X1	2 (3.2)	-	-	2 (1.0)
X3	44 (71.0)	9 (19.1)	3 (3.4)	56 (28.3)
X4	-	-	2 (2.2)	2 (1.0)
X3 and X4	3 (4.8)	1 (2.1)	-	4 (2.0)
Total	49 (79.0)	10 (21.3)^b^	5 (5.6)	64 (32.3)

*^*a*^No X2 and X5 replicons were detected. ^*b*^X3 replicon was detected in isolates producing OXA-181 (*n* = 5), OXA-232 (*n* = 4), and KPC-2 (*n* = 1). One of the OXA-181 producers was also positive for the X4 subtypes*.

### Characteristics of the NDM-Producing Isolates Carrying IncX3 Plasmids

The 47 NDM-producing isolates carrying IncX3 plasmids were investigated further. The species of the isolates include 27 *E. coli*, 11 *K. pneumoniae*, 3 *E. cloacae*, 3 *E. aerogenes*, 2 *C. freundii* and 1 *K. oxytoca*. PCR and sequencing showed that the isolates had *bla*_NDM-1_ (*n* = 11), *bla*_NDM-5_ (*n* = 35), and *bla*_NDM-7_ (*n* = 1). In all the isolates, hybridization localized the *bla*_NDM_ gene to the IncX3 plasmids. All the IncX3 plasmids had size of approximately 50 kb. A wide diversity of sequence types (STs) were found among the *E. coli* and *K. pneumoniae* isolates. Among the 27 *E. coli* isolates, 22 different STs were identified. Two isolates each belonged to ST10, ST48, ST167, ST410, and ST744. The other isolates (*n* = 17) belong to single diverse STs (including ST156, ST224, ST354, ST522, ST617, ST641, ST718, ST761, ST877, ST964, ST1101, ST1147, ST2973, ST5229, and three new STs). The three new STs included a single locus variant (SLV) of ST10 (profile for *adk-fumC-gyrB-icd-mdh-purA-recA*, 605-11-4-8-8-8-2), a SLV of ST7122 (profile 623-11-4-12-8-new) and a ST with three new alleles (profile 87-new-136-new-new-1-157) (**Supplementary Figure S1**). Among the 11 *K. pneumoniae* isolates, 10 different STs were identified including ST34 (*n* = 2) and nine single isolate STs (ST48, ST197, ST399, ST438, ST629, ST672, and three new STs). The three new STs included ST3386, ST3387, and ST3388. All except one *E. coli* isolate had ertapenem, imipenem, and meropenem MICs in the intermediate or resistant range (**Table 2**). The only meropenem-sensitive isolate was intermediate to ertapenem and imipenem. Susceptibility rates were high (>90%) for amikacin, colistin, and tigecycline. Co-resistance to levofloxacin, gentamicin, and minocycline were common.

**Table 2:** Antimicrobial susceptibilities of the 47 NDM-producing isolates carrying IncX3 plasmids.

Agent	%			MIC_50_/MIC_90_ (μg/ml)	MIC range (μg/ml)
	S	I	R		
Ertapenem	0	0	100	≥16/≥16	2 to ≥16
Imipenem	0	2.1	97.8	≥16/≥16	2 to ≥16
Meropenem	2.1	2.1	95.8	≥16/≥16	1 to ≥16
Levofloxacin	51.1	2.1	46.8	2/≥8	≤0.12 to ≥8
Gentamicin	59.6	0	40.4	≤1/≥16	≤1 to ≥16
Minocycline	68.1	17.0	14.9	2/≥16	≤1 to ≥16
Colistin	93.6	0	6.4	≤0.25/0.5	≤0.25 to >4
Amikacin	95.7	0	4.3	≤4/≤4	≤4 to ≥64
Tigecycline	97.9	2.1	0	≤1/≤1	≤1 to 2

*S, sensitive; I, intermediate; R, resistant. Colistin and tigecycline were interpreted according to EUCAST v8.1. All other drugs were interpreted according to 2018 CLSI M100*.

**FIGURE 1:**
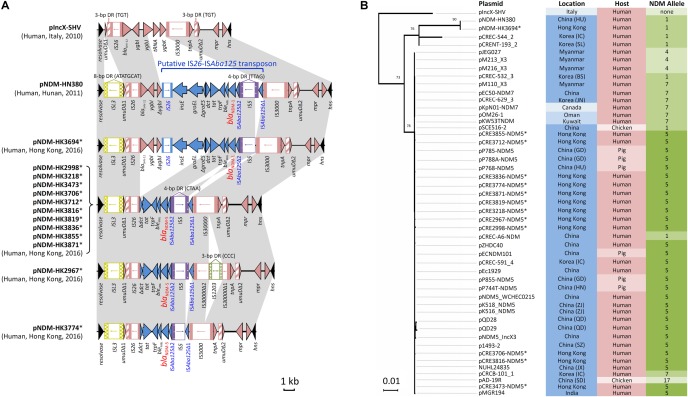
Analysis of IncX3 plasmid sequences in this study. **(A)** Comparison of genetic load regions in 13 plasmids harboring *bla*_NDM_ with two reference plasmids (pIncX-SHV and pNDM-HN380). **(B)** Phylogenetic analysis of 49 IncX3 plasmids using the Maximum Likelihood method based on the Tamura-Nei model. These included two reference plasmids, 13 plasmids in this study and 34 plasmids identified in GenBank (last accessed 16 May, 2018). Branches were drawn to scale, with lengths measured in the number of substitutions per site. Plasmids sequenced in the present study are marked with an asterisk. Each plasmid was labeled and color-coded by the geographical origin (blue intensity: high, Asia; moderate, Middle East; low, Europe and North America), host source (pink intensity: high, human; low, animals), and NDM allele (green intensity: high, allele 5; moderate, alleles 1 and 7; low, alleles 4 and 17).

Complete sequences of 13 *bla*_NDM_-carrying IncX3 plasmids with sizes of ∼46 kb were obtained (**Supplementary Table S2**). This subset of isolates was randomly chosen from among the 47 NDM-producing isolates with IncX3 plasmids, including isolates from hospitals in six different healthcare regions. Twelve plasmids carried *bla*_NDM-5_ and one plasmid carried *bla*_NDM-1_. They have a plasmid scaffold typical of IncX3 plasmids. The genetic load regions in the plasmids were compared with two reference IncX3 plasmids (pIncX-SHV and pNDM-HN380) (**Figure 1A**). Features shared among the *bla*_NDM_-carrying plasmids include: (a) an IS*L3* with 8-bp flanking direct repeats (ATATGCAT) downstream of the resolvase gene; and (b) the *umuD* gene was split into two fragments (*umuDΔ*1 and *umuDΔ2*) at the same position resulting in a pair of 3-bp direct repeats (TGT). In the plasmids, *bla*_NDM_ was inserted as a putative IS*26*-IS*Aba125* transposon. The genetic load region in one plasmid carrying *bla*_NDM-1_ (pNDM-HK3694) was virtually identical to that in pNDM-HN380, except for a small deletion downstream of the IS*3000*. In ten *bla*_NDM-5_-carrying plasmids originating from different species (5 *E. coli*, 4 *K. pneumoniae*, 1 *E. cloacae*) and six hospitals, the sequences inserted between the two *umuD* fragments were 100% identical (10117 bp in length). This inserted sequence differs from that in pNDM-HN380 by a deletion of 7874 bp (**Figure 1A**). The remaining two plasmids exhibited one or two minor variations including an IS*1203* inserted within IS*3000* (pNDM-HK2967) or an additional deletion (616 bp) at the junction between the IS*5* and IS*Aba125*Δ1 remnant (pNDM-HK3774). In all the 12 NDM-5 plasmids, IS*5* was inserted at the same position leading to the flanking 4-bp direct repeats (CTAA). In the two NDM-1 plasmids (pNDM-HK3694 and pNDM-HN380), IS*5* was inserted at a different position in the opposite orientation.

Next, the complete sequence of pNDM-HK2998 (chosen as a representative) was used to query the GenBank database. This allowed identification of another 34 plasmids related to pNDM-HK2998 (**Figure 1B**), including 30 from Asia (China, Korea, Myanmar, India), two from Middle East countries (Oman, Kuwait), and one each from Italy and Canada. This comprised plasmids originating from human (*n* = 26) and food animals (*n* = 8). Multiple NDM variants (4, 5, 7, and 17) which differ from NDM-1 by one or more amino acids (V88L, D130N, M154L, and E170K) were carried by the plasmids. Alignment and phylogenetic analysis showed that the plasmids were highly similar in both the backbone and genetic load regions (**Figure 1B**).

### Host Range and Conjugation Frequencies

**FIGURE 2:**
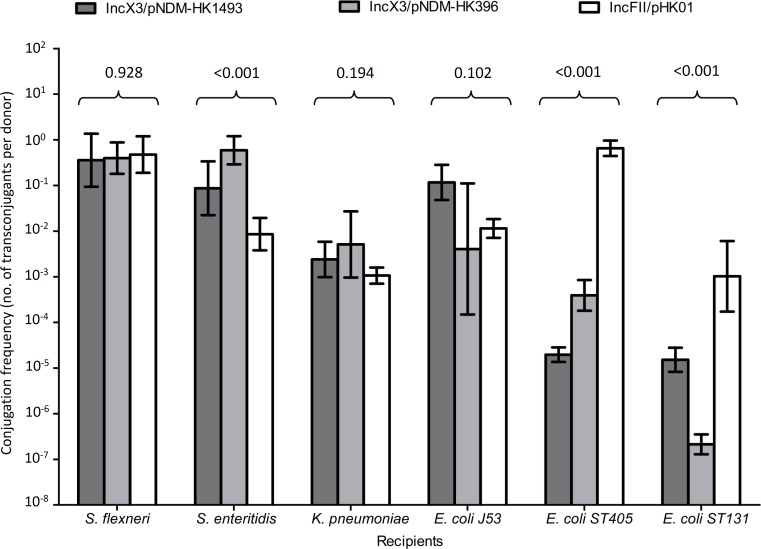
Conjugation frequencies of IncX3 and IncFII plasmids to six recipients of different species. The donor *E. coli* strains for the IncX3 plasmids (pNDM-HK1493 and pNDM-HK396) were CRE1493 and CRE396, respectively, while for the IncFII (pHK01) plasmid was combat2D2. The histograms show means and standard deviations (error bars). Conjugation frequencies were logarithmically transformed prior to statistical analysis.

In conjugation experiments, the IncX3 and IncFII plasmids could be successfully transferred from the original clinical strains to the *E. coli, S. flexneri, S. enteritidis*, and *K. pneumoniae* recipients (**Figure 2**). The highest frequencies of conjugative transfer of the IncX3 plasmids were observed for the *S. flexneri* and *S. enteritidis* recipients (≥10^-1^ per donor cell), following by the *K. pneumoniae* recipient (10^-2^ to 10^-3^ per donor cell). These conjugative frequencies were comparable or higher than those for the reference IncFII plasmid. Conjugation frequencies of the IncX3 plasmids in the *E. coli* recipients were variable; higher in the J53 recipient than in the two recipients of ST405 and ST131 lineages. In the latter two recipients, conjugation frequencies of the two IncX3 plasmids were lower than those for the reference IncFII plasmid. Despite repeated attempts, conjugative transfer of the IncX3 plasmids to *P. aeruginosa* and *A. baumannii* recipients was not successful.

**FIGURE 3:**
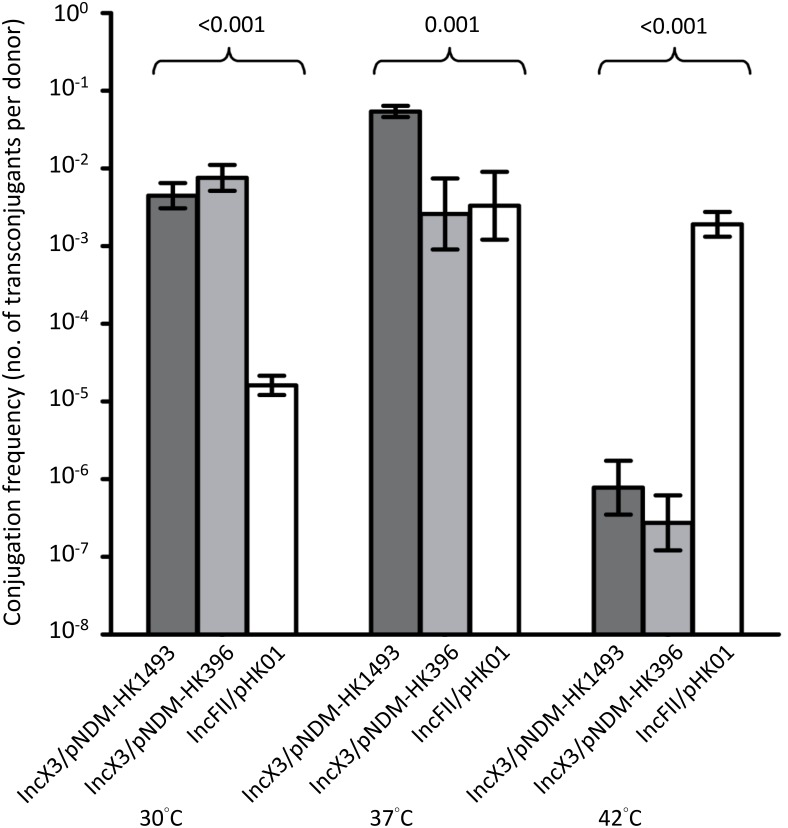
Conjugation frequencies of IncX3 and IncFII plasmids at three temperatures. The donor *E. coli* strains for the IncX3 plasmids (pNDM-HK1493 and pNDM-HK396) were CRE1493 and CRE396, respectively, while for the IncFII plasmid (pHK01) was combat2D2. The histograms show means and standard deviations (error bars). Conjugation frequencies were logarithmically transformed prior to statistical analysis.

Incubation temperatures had different effects on the transfer frequencies of the IncX3 and IncFII plasmids (**Figure 3**). At 30 and 37°C, conjugation frequencies of the IncX3 plasmids were comparable or higher (by 10^1^–10^3^-fold) than those for the reference IncFII plasmid. Incubation at 42°C inhibited the conjugative transfer of the IncX3 plasmids but not the IncFII plasmid.

## Discussion

This study demonstrated the importance of pNDM-HN380-like, IncX3 plasmids in the dissemination of *bla*_NDM_ among bacterial isolates of multiple *Enterobacteriaceae* species in Hong Kong. These plasmids can be considered epidemic because they have previously been detected in multiple countries and among bacteria of human and animal host sources (Ho et al., 2012b, 2018a; Wailan et al., 2015; Petrosillo et al., 2016; Choudhury et al., 2018). Our isolates carrying IncX3 plasmids were mostly recovered from patients who have history of previous hospitalization in a healthcare institute either locally or in mainland China. In the collection, approximately three-quarters of them were rectal swab isolates which were detected through active surveillance culture upon hospital admission (Ho et al., 2011). It is likely that hospitals are the main reservoirs of these *bla*_NDM_ carrying plasmids. Additional reservoirs or sources of this resistance mechanism include commensals carried by food animals or household contacts of the patients (Ho et al., 2012a, 2018a). In China, similar IncX3 plasmids carrying *bla*_NDM_ have been detected in *E. coli* from swine in Guangdong, Henan, and Hunan provinces and from a chicken in Shandong Province (Liu et al., 2017; Ho et al., 2018a).

A high degree of synteny was demonstrated among the complete plasmid sequences in the present analysis. With the exception of a few insertions and deletions, insertion sequence elements bearing identical direct repeats and other genes within the genetic load regions harboring *bla*_NDM_ were shared between IncX3 plasmids from geographically widespread regions and host sources (**Figure 1**). The finding points to a single *bla*_NDM_ insertion into a common IncX3 plasmid ancestor (pNDM-HN380-like), rather than multiple, independent *bla*_NDM_ insertions in the IncX3 platform. Variants of *bla*_NDM-1_ then arise through single nucleotide substitution in the *bla*_NDM_ coding region. The NDM-1 variants that were identified in IncX3 plasmids including NDM-4, NDM-5, NDM-7, and NDM-17 have enhanced carbapenemase activity, leading to increased isolate resistance to carbapenem (Makena et al., 2015; Liu et al., 2017).

We showed that the IncX3 plasmids carrying *bla*_NDM_ could be transferred to different bacterial species at frequencies that are comparable or higher than the epidemic IncFII plasmid carrying *bla*_CTX-M_. The efficient transfer of *bla*_NDM_ plasmids to two clinical isolates of *S. flexneri* and *S. enteritidis* is concerning as this will render all the beta-lactam antibiotics ineffective. The conjugal transfer of the IncX3 plasmids carrying *bla*_NDM_ to recipient *E. coli* over a range of temperature is also of note. The finding has implications for the dissemination of *bla*_NDM_ in many environmental sources such as abiotic touch surface, sewage, waterway, and soil in tropical countries as well as in the guts of mammalian and avian species (Walsh et al., 2011; Warnes et al., 2012). In many Asian and Middle East countries, the ambient temperature in the summer is often above 30°C. Hence, this plasmid type may be more effective than the other plasmid types in the environmental dissemination of *bla*_NDM_ (Walsh et al., 2011). Interestingly, the conjugal transfer of a related subtype, IncX4 at 30°C to recipient *E. coli* is also higher than IncFII, suggesting that this may be a property shared by all the subtypes of IncX plasmid (Lo et al., 2014).

## Conclusion

This study identified a highly conjugative, pNDM-HN380-like IncX3 plasmid to be a major vehicle for dissemination of *bla*_NDM_ among multiple enterobacterial species in Hong Kong hospitals. Analysis of complete plasmid sequences confirms that this epidemic NDM plasmid is widespread in the ecosystem.

## Accession Numbers

The complete sequences of the 13 IncX3 plasmids were deposited in the GenBank database under accession numbers MH234497 (pNDM-HK3871), MH234498 (pNDM-HK3855), MH234499 (pNDM-HK3836), MH234500 (pNDM-HK3819), MH234501 (pNDM-HK3816), MH234502 (pNDM-HK3774), MH234503 (pNDM-HK3712), MH234504 (pNDM-HK3706), MH234505 (pNDM-HK3694), MH234506 (pNDM-HK3473), MH234507 (pNDM-HK3218), MH234508 (pNDM-HK2998), and MH234509 (pNDM-HK2967).

## Author Contributions

P-LH and YW conceived and designed the experiments and wrote the paper. P-LH, VC, CT, AW, RL, W-KL, and DT collected the bacteria and related the data. YW, M-KT, and K-HC performed the experiments. P-LH, K-HC, and YW analyzed the data. All authors provide critical input to the manuscript and endorsed the final version.

## Conflict of Interest Statement

The authors declare that the research was conducted in the absence of any commercial or financial relationships that could be construed as a potential conflict of interest.

## Footnotes

1 https://www-is.biotoul.fr/about.php
