# Supplementary material for: Occurrence of Highly Conjugative IncX3 Epidemic Plasmid Carrying blaNDM in Enterobacteriaceae Isolates in Geographically Widespread Areas
Source: Front Microbiol. 2018 Sep 20;9:2272. doi: 10.3389/fmicb.2018.02272 (PMC6158458; doi:10.3389/fmicb.2018.02272)
Supplement: Supplementary file 1 [file Data_Sheet_1.pdf]

Supplementary file

Table S1. Culture medium and antibiotics used for selection of transconjugants

Recipient strain and feature	Agar medium	Agent or antibiotic added to agar medium in conjugation experiment[a]	
		NDM-carrying IncX3 plasmids	CTX-M-14-carrying pHK01 plasmid
Escherichia coli J53<br>(azide-R, laboratory strain)	MacConkey	Azide and meropenem	Azide and cefotaxime
E. coli ST405<br>(Azi-R, clinical isolate)	MacConkey	Azide and meropenem	Azide and cefotaxime
E. coli ST131<br>(Azi-R, clinical isolate)	MacConkey	Azide and meropenem	Azide and cefotaxime
Shigella flexneri<br>(clinical isolate)	XLD	Meropenem	Cefotaxime
Salmonella enteritidis<br>(clinical isolate)	XLD	Meropenem	Cefotaxime
Klebsiella pneumoniae<br>(clinical isolate)	UriSelect4	Meropenem	Cefotaxime
Pseudomonas aeruginosa<br>(Azi-R, PT224, ATCC 27853, NCTC 10662)	MacConkey	Azide and meropenem	Azide and cefotaxime
Acinetobacter baumannii<br>(Azi-R, ATCC 17959, ATCC 19606)	MacConkey	Azide and meropenem	Azide and cefotaxime

Abbreviaitons: Azi-R, resistant to sodium azide; XLD, xylose Lysine Deoxycholate.

[a] At the following concentration: sodium azide (100 µg ml^{-1}), meropenem (0.25 µg ml^{-1}) and/or cefotaxime (1 µg ml^{-1}).

Table S2. Summary of 49 IncX3 plasmids.

No.	Plasmid Name[a]	NDM allele	Geographical Origin[b]	Species[c]	Accession	Plasmid size, bp (query coverage/identity)[d]	Host source	Year collected	Genetic load region, bp[e]
1	pIncX-SHV	None	Italy	KP	JN247852	43,380 (94.0%/99.9%)	Human	2010	13,287
2	pNDM-HN380	NDM-1	China (HU)	KP	JX104760	54,035 (100.0%/99.9%)	Human	2011	23,944
3	pCRE3694-NDM1*	NDM-1	Hong Kong	EC	MH234505	53,097 (98.7%/99.9%)	Human	2016	23,006
4	pCREC-544_2	NDM-1	Korea (IC)	EC	CP024828	51,455 (100.0%/99.9)	Human	2015	21,365
5	pCRENT-193_2	NDM-1	Korea (SL)	ENTSP	CP024814	44,962 (97.4%/99.9%)	Human	2013	14,871
6	pJEG027	NDM-4	Myanmar	KP	KM400601	46,253 (100.0%/99.8%)	Human	na	16,070
7	pM213_X3	NDM-4	Myanmar	EC	AP018142	43,534 (94.0%/99.9%)	Human	2015	13,443
8	pM216_X3	NDM-4	Myanmar	EC	AP018146	46,161 (100.0%/99.9%)	Human	2015	16,070
9	pCREC-532_3	NDM-1	Korea (BS)	EC	CP024833	46,137 (99.9%/99.9%)	Human	2015	16,070
10	pM110_X3	NDM-7	Myanmar	EC	AP018141	45,122 (97.7%/99.9%)	Human	2015	15,031
11	pEC50-NDM7	NDM-7	China	EC	KX470735	46,161 (100.0%/99.9%)	Human	na	16,070
12	pCREC-629_3	NDM-7	Korea (JN)	EC	CP024818	46,161 (100.0%/99.9%)	Human	2015	16,070
13	pKpN01-NDM7	NDM-7	Canada	KP	CP012990	46,161 (100.0%/99.9%)	Human	2013	16,070
14	pOM26-1	NDM-7	Oman	EC	KP776609	45,122 (97.7%/99.9%)	Human	na	15,031
15	pKW53TNDM	NDM-7	Kuwait	EC	KX214669	46,161 (100.0%/99.9%)	Human	2012	16,070
16	pSCE516-2	NDM-1	China	EC	KX023261	46,254 (100.0%/99.9%)	Chicken	2015	16,071
17	pCRE3855-NDM5*	NDM-5	Hong Kong	EC	MH234498	46,161 (100.0%/100.0%)	Human	2016	16,070
18	pCRE3712-NDM5*	NDM-5	Hong Kong	EC	MH234503	46,161 (100.0%/100.0%)	Human	2016	16,070
19	pP785-NDM5	NDM-5	China (GD)	EC	MF547509	46,161 (100.0%/99.9%)	Pig	2016	16,070
20	pP788A-NDM5	NDM-5	China (GD)	EC	MF547510	46,161 (100.0%/100.0%)	Pig	2016	16,070
21	pP768-NDM5	NDM-5	China (HU)	EC	MF547508	46,161 (100.0%/100.0%)	Pig	2016	16,070
22	pCRE3836-NDM5*	NDM-5	Hong Kong	EC	MH234499	46,161 (100.0%/99.9%)	Human	2016	16,070
23	pCRE3774-NDM5*	NDM-5	Hong Kong	EC	MH234502	45,547 (98.7%/100.0%)	Human	2016	15,456
24	pCRE3871-NDM5*	NDM-5	Hong Kong	EC	MH234497	46,161 (100.0%/100.0%)	Human	2016	16,070
25	pCRE3819-NDM5*	NDM-5	Hong Kong	KP	MH234500	46,161 (100.0%/100.0%)	Human	2016	16,070
26	pCRE3218-NDM5*	NDM-5	Hong Kong	EC	MH234507	46,161 (100.0%/100.0%)	Human	2016	16,070
27	pCRE2967-NDM5*	NDM-5	Hong Kong	EC	MH234509	47,474 (100.0%/99.9%)	Human	2016	17,383
28	pCRE2998-NDM5*	NDM-5	Hong Kong	KP	MH234508	46,161 (100.0%/100.0%)	Human	2016	16,070
29	pCREC-A6-NDM	NDM-1	China	EC	KX960109	46,037 (100.0%/99.7%)	Human	na	16,002
30	pZHDC40	NDM-5	China	EC	KY041843	45,346 (98.2%/100.0%)	Human	2015	15,255

31	pECNDM101	NDM-5	China	EC	KX507346	46,165 (100.0%/99.9%)	Pig	na	16,073
32	pCREC-591_4	NDM-5	Korea (IC)	EC	CP024825	46,161 (100.0%/99.9%)	Human	2015	16,070
33	pEc1929	NDM-5	China	EC	KT824791	46,164 (100.0%/99.9%)	Human	2014	16,071
34	pP855-NDM5	NDM-5	China (GD)	EC	MF547511	46,161 (100.0%/99.9%)	Pig	2017	16,070
35	pP744T-NDM5	NDM-5	China (HN)	EC	MF547507	45,547 (98.7%/100.0%)	Pig	2015	15,456
36	pNDM5_WCHEC0215	NDM-5	China	EC	KY435936	47,337 (100.0%/97.4%)	Human	na	17,268
37	pK518_NDM5	NDM-5	China (ZJ)	KM	CP023188	46,146 (100.0%/99.9%)	Human	2017	16,070
38	pK516_NDM5	NDM-5	China (ZJ)	KM	CP022351	46,149 (100.0%/99.9%)	Human	2017	16,070
39	pNDM-QD28	NDM-5	China (QD)	EC	KU167608	46,161 (100.0%/99.9%)	Human	2013	16,070
40	pNDM-QD29	NDM-5	China (QD)	EC	KU167609	46,161 (100.0%/99.9%)	Human	2013	16,070
41	pNDM5_IncX3	NDM-5	China	KP	KU761328	46,161 (100.0%/99.9%)	Human	2015	16,070
42	p1493-2	NDM-5	China (SZ)	EC	CP019073	46,145 (100.0%/99.9%)	Human	2013	16,068
43	pCRE3706-NDM5*	NDM-5	Hong Kong	KP	MH234504	46,161 (100.0%/99.9%)	Human	2016	16,070
44	pCRE3816-NDM5*	NDM-5	Hong Kong	KP	MH234501	46,161 (100.0%/99.9%)	Human	2016	16,070
45	NUHL24835	NDM-5	China (JX)	KP	CP014006	46,161 (100.0%/99.9%)	Human	2014	16,070
46	pCRCB-101_1	NDM-7	Korea (IC)	CF	CP024820	46,161 (100.0%/99.9%)	Human	2015	16,070
47	pAD-19R	NDM-17	China (SD)	EC	KX833071	46,161 (100.0%/99.9%)	Chicken	2015	16,070
48	pCRE3473-NDM5*	NDM-5	Hong Kong	ECLO	MH234506	46,161 (100.0%/100.0%)	Human	2016	16,070
49	pNDM-MGR194	NDM-5	India	KP	KF220657	46,253 (100.0%/99.9%)	Human	2011-13	16,070

[a] Plasmids sequenced in the present study are marked with an asterisk

[b]BS, Busan; IC, Incheon ; SL, Seoul; GD, Guangdong; BJ, Beijing; HN, Henan; HU, Hunan; JN, Jeonnam; JS, Jiangsu; JX, Jiangxi; QD, Qingdao; SD, Shandong; ZJ, Zhejiang.

[c] CF, *Citrobacter freundii*; EC, *Escherichia coli*; ECLO, *Enterobacter cloacae*; ENTSP, *Enterobacter species*; KP, *Klebsiella pneumoniae*; KM, *Klebsiella michiganensis*

[d] Percentages coverage and identity by using pNDM-HK2998 as a reference

[e] The genetic load region is between the resolvase gene and the *hns* gene. In all the plasmids, IS*L3* was inserted at the same positions in the same orientations.

Figure S1. Sequence alignment of new *fumC*, *icd*, *mdh* and *recA* alleles in two *E. coli* strains (CRE 3725 and CRE 3775) from this study with other closely related alleles.

fumC

```
fumC251      ------------------------------------------------------------   60
fumC71       ------------------------------------------------------------   60
fumC709      ------------------------------------------------------------   60
fumC385      -------------G----------------------------------------------   60
CRE3725      CGAGCGCCATTCGTCAGGCGGCGGATGAAGTACTGGCAGGACAGCATGACGACGAATTCC   60
                          14
fumC251      ----------------------------------------T-------------------   120
fumC71       ------------------------------------------------------------   120
fumC709      ------------------------------------------------------------   120
fumC385      ------------------------------------------------------------   120
CRE3725      CGCTGGCTATCTGGCAGACCGGCTCCGGCACGCAAAGTAACATGAACATGAACGAAGTGC   120
                                                    101

fumC251      -------T-----------------T----------------------------------   180
fumC71       -------------------------------T----------------------------   180
fumC709      -------------------------------T----------------------------   180
fumC385      ------------------------------------------------------------   180
CRE3725      TGGCTAACCGGGCCAGTGAATTACTCGGCGGCGTGCGCGGGATGGAACGTAAAGTTCACC   180
                    128               146   152

fumC251      -------C----------------------------------------------------   240
fumC71       -------C--------------------C-------------------------------   240
fumC709      ---------------------------------------------------------G--   240
fumC385      ------------------------------------------------------------   240
CRE3725      CTAACGATGACGTGAACAAAAGCCAAAGTTCCAACGATGTCTTTCCGACGGCGATGCACG   240
                    188                  209                          238

fumC251      ------------------------------------------------------------   300
fumC71       ------------------------------------------------------------   300
fumC709      ------------------------------------------------------------   300
fumC385      ------------------------------------------------------------   300
CRE3725      TTGCGGCGCTGCTGGCGCTGCGCAAGCAACTCATTCCGCAGCTTAAAACCCTGACACAGA   300

fumC251      -G------------------A---------------------------------------   360
fumC71       ------------------------------------------------------------   360
fumC709      ------------------------------------------------------------   360
fumC385      ------------------------------------------------------------   360
CRE3725      CACTGAATGAGAAATCCCGTGCATTTGCCGATATCGTCAAAATTGGTCGTACTCACTTGC   360
              302                321


fumC251      -------------------G-----------------------------------G----   420
fumC71       -------------------G----------------------------------------   420
fumC709      -------------------G-----------------------------------T----   420
fumC385      -------------------A----------------------------------------   420
CRE3725      AGGATGCCACGCCGTTAACTCTGGGGCAGGAGATTTCCGGCTGGGTAGCGATGCTCGAGC   420
                                380                                416

fumC251      ----------------T--------------------------------       469
fumC71       -------------------------------------------------       469
fumC709      -------------------------------------------------       469
fumC385      -------------------------------------------------       469
CRE3725      ATAATCTCAAACATATCGAATACAGCCTGCCTCACGTAGCGGAACTGGC       469
                             437
```

icd

```
icd161    ------------------------------------------------------------   60
icd479    ------------------------------------------------------------   60
icd242    ------------------------------------------------------------   60
icd168    ------------------------------------------------------------   60
CRE3725   CGACGCTGCAGTCGAGAAAGCCTATAAAGGCGAGCGTAAAATCTCCTGGATGGAAATTTA   60

icd161    ------------------------------------------------------------   120
icd479    ------------------------------------------------------------   120
icd242    ------------------------------------------------------------   120
icd168    ------------------------------------------------------------   120
CRE3725   CACCGGTGAAAAATCCACACAGGTTTATGGTCAGGATGTCTGGCTGCCTGCTGAAACCCT   120

icd161    ------------------------------------------------------------   180
icd479    ------------------------------------------------------------   180
icd242    ------------------------------------------------------------   180
icd168    ------------------------------------------------------------   180
CRE3725   TGATCTGATTCGTGAATATCGCGTTGCTATTAAAGGCCCGCTGACCACTCCGGTTGGTGG   180

icd161    ---------------G--------------------------------------------   240
icd479    ---------------C--------------------------------------------   240
icd242    ---------------G--------------------------------------------   240
icd168    ------------------------------------------------------------   240
CRE3725   CGGTATTCGTTCTCTTAACGTTGCCCTGCGCCAGGAACTGGATCTCTACATCTGCCTGCG   240
                        196

icd161    ------------------------------------------------------------   300
icd479    ------------------------------------------------------------   300
icd242    ------------------------------------------------------------   300
icd168    ------------------------------------------------------------   300
CRE3725   TCCGGTACGTTACTATCAGGGCACCCCAAGCCCGGTTAAACACCCTGAACTGACCGATAT   300

icd161    ------------------------------------------------------------   360
icd479    ------------------------------------------------------------   360
icd242    -----------------------------------G------------------------   360
icd168    ------------------------------------------------------------   360
CRE3725   GGTTATCTTCCGTGAAAACTCGGAAGACATTTATGCAGGTATCGAATGGAAAGCTGACTC   360
                                            336

icd161    ------------------------------------------------------------   420
icd479    ------------------------------------------------------------   420
icd242    ---------C--------------------------------------------------   420
icd168    ------------------------------------------------------------   420
CRE3725   TGCCGACGCTGAGAAAGTGATTAAATTCCTGCGTGAAGAGATGGGCGTGAAGAAAATTCG   420
                  370

icd161    ---------------C-----------C--------------------------------   480
icd479    ---------C-----------------------------A-----------T--------   480
icd242    ---------------------------------------G--------------------   480
icd168    ---------------------------------------G--------------------   480
CRE3725   CTTCCCGGAACATTGTGGTATCGGTATTAAGCCGTGTTCTGAAGAAGGCACCAAACGTCT   480
                  430   436         448         460         472

icd161    ---C-----C----------------------------   518
icd479    --------------------------------------   518
icd242    --------------------------------------   518
icd168    --------------------------------------   518
CRE3725   GGTTCGTGCAGCGATCGAATACGCAATTGCTAACGATC   518
            484   490
```

mdh

```
mdh590        ------------------------------------------------------------   60
mdh441        --T---------------------------------------------------------   60
mdh249        ------------------------------------------------------------   60
mdh6          -----------------G------------------------------------------   60
CRE3725       GGCGTAGCGCGTAAACCAGGTATGGATCGTTCCGACCTGTTTAACGTTAACGCCGGCATC   60
                3              18

mdh590        -----------------------------------------------------------T   120
mdh441        ------------------------------------------------------------   120
mdh249        ------------------------------------------------------------   120
mdh6          ------------------------------------------------------------   120
CRE3725       GTGAAAAACCTGGTACAGCAAGTTGCGAAAACCTGCCCGAAAGCGTGCATTGGTATTATC   120
                                                                        120

mdh590        --------------------------G---------------------------------   180
mdh441        ------------------------------------------------------------   180
mdh249        --------------------T---------------------------------------   180
mdh6          ------------------------------------------------------------   180
CRE3725       ACTAACCCGGTTAACACCACAGTTGCAATTGCTGCTGAAGTGCTGAAAAAAGCCGGTGTT   180
                                  141   147
mdh590        ------------------------------------------------------------   240
mdh441        ------------------------------------------------------------   240
mdh249        ------------------------------------------------------------   240
mdh6          ------------------------------------------------------------   240
CRE3725       TATGACAAAAACAAACTGTTCGGCGTTACCACGCTGGATATCATTCGTTCCAACACCTTT   240

mdh590        ------------------------------------------------------------   300
mdh441        ------------------------------------------------------------   300
mdh249        ------------------------------------------------------------   300
mdh6          ------------------------------------------------------------   300
CRE3725       GTTGCGGAACTGAAAGGCAAACAGCCAGGCGAAGTTGAAGTGCCGGTTATTGGCGGTCAC   300

mdh590        ------------------------------------------------------------   360
mdh441        ------------------------------------------------------------   360
mdh249        ------------------------------------------------------------   360
mdh6          ------------------------------------------------------------   360
CRE3725       TCTGGTGTTACCATTCTGCCGCTGCTGTCACAGGTTCCTGGCGTTAGTTTTACCGAGCAG   360

mdh590        ------------------------------------------------------------   420
mdh441        ------------------------------------------------------------   420
mdh249        ------------------------------------------------------------   420
mdh6          ------------------------------------------------------------   420
CRE3725       GAAGTGGCTGATCTGACCAAACGTATCCAGAACGCGGGTACTGAGGTGGTTGAAGCGAAA   420

mdh590        -------------------------------- 452
mdh441        -------------------------------- 452
mdh249        -------------------------------- 452
mdh6          -------------------------------- 452
CRE3725       GCCGGTGGCGGGTCTGCAACCCTGTCTATGGG 452
```

recA

```
recA380         ---------------------------T--------------------------------   60
recA221         T-----------------------------------------------------------   60
recA2           ------------------------------------------------------------   60
recA490         ------------------------------------------------------------   60
CRE3775         CGCACGTAAACTGGGCGTCGATATCGACAACCTGCTGTGCTCCCAGCCGGACACCGGCGA   60
                1                          28

recA380         ------------------------------------------------------------   120
recA221         ------------------------------------------------------------   120
recA2           ------------------------------------------------------------   120
recA490         ------------------------------------------------------------   120
CRE3775         GCAGGCACTGGAAATCTGTGACGCCCTGGCGCGTTCTGGCGCAGTAGACGTTATCGTCGT   120

recA380         ------------------------------------------------------------   180
recA221         ---------------------------------------------------C--------   180
recA2           ---------------------------------------------------C--------   180
recA490         ---------------------------------------------------A--------   180
CRE3775         TGACTCCGTGGCGGCACTGACGCCGAAAGCGGAAATCGAAGGCGAAATCGGTGACTCTCA   180
                                                  172
recA380         ------------------------------------------------------------   240
recA221         ------------------------------------------------------------   240
recA2           ------------------------------------------------------------   240
recA490         ------------------------------------------------------------   240
CRE3775         CATGGGCCTTGCGGCACGTATGATGAGCCAGGCGATGCGTAAGCTGGCGGGTAACCTGAA   240

recA380         ------------------------------------------------------------   300
recA221         ------------------------------------------------------------   300
recA2           ------------------------------------------------------------   300
recA490         ------------------------------------------------------------   300
CRE3775         GCAGTCCAACACGCTGCTGATCTTCATCAACCAGATCCGTATGAAAATTGGTGTGATGTT   300

recA380         ------------------------------------------------------------   360
recA221         ------------------------------------------------------------   360
recA2           ------------------------------------------------------------   360
recA490         ------------------------------------------------------------   360
CRE3775         CGGTAACCCGGAAACCACTACCGGTGGTAACGCGCTGAAATTCTACGCCTCTGTTCGTCT   360

recA380         ------------------------------------------------------------   420
recA221         ------------------------------------------------------------   420
recA2           ------------------------------------------------------------   420
recA490         ------------------------------------------------------------   420
CRE3775         CGACATCCGTCGTATCGGCGCGGTGAAAGAGGGCGAAAACGTGGTGGGTAGCGAAACCCG   420

recA380         ------------------------------------------------------------   480
recA221         ------------------------------------------------------------   480
recA2           ------------------------------------------------------------   480
recA490         ------------------------------------------------------------   480
CRE3775         CGTGAAAGTGGTGAAGAACAAAATCGCTGCGCCGTTTAAACAGGCTGAATTCCAGATCCT   480

recA380         ------------------------------   510
recA221         ------------------------------   510
recA2           ------------------------------   510
recA490         ------------------------------   510
CRE3775         CTACGGCGAAGGTATCAACTTCTACGGCGA   510
```
